# 

**Published:** 1856-12

**Authors:** 


### CONTENTS.

ORIGINAL ESSAYS AND COMMUNICATIONS.

Dental Wreath—Original Poem ........ 153
Dr. Slayton's Gutta Percha ........ 167
Essay upon Professional Fees. By ELISHA TOWNSEND, D. D. S., M. D., ........ 169
Dental Chemistry of the Mouth. By GEO. WATT ........ 180
The Exciting Causes of Caries. By J. TAFT ........ 187
Random Thoughts ........ 192
One Idea ........ 195
Chapman's Introductory Lecture ........ 198
Dental Convention ........ 215
Plate Forceps ........ 217
Impressions. By H. R. SMITH, D. D. S., M. D. ........ 218
Mode of Inserting Partial Sets. By F. A. BREWER ........ 224
Dental Periodicals ........ 325
Classification of Decayed Cavities in the Teeth. By J. TAFT ........ 230

SELECTED ARTICLES.

An Improved Method of Using Gold Foil. By PROF. ARTHUR ........ 232
Amalgam. By J. D. WHITE ........ 240
Sensitive Dentine. By J. H. M'QUILLEN ........ 243
Ergot and Digitalis in Hemorrhage ........ 248
Public Meeting of the Dental Profession ........ 248

NOTICES OF EXCHANGES ........ 249

The Ohio Medical and Surgical Journal—The Peninsular Journal of Medicine and the Collateral Sciences—Charleston Medical Journal and Review—The St. Louis Medical and Surgical Journal—The Boston Medical and Surgical Journal—The Memphis Medical Recorder—The Western Lancet—The Cincinnati Medical Observer—The Inventor—Our Exchanges.

MISCELLANEOUS ........ 252

Questions and Answers—Infirmary Report of the Ohio College of Dental Surgery—Report of the Executive Committee of the Mississippi Valley Association of Dental Surgeons—Tooth within a Tooth.

EDITORIAL ........ 256

The Ohio College of Dental Surgery—Our Last Number—Brown's Dental Depot—Palmer's Dental Depot—Advertisement of Messrs. Thorne & Co.—Toland's Dental Depot—Handles of Plugging Instruments—Mississippi Valley Association—Proceedings of the Dental Convention—Ohio Dental College Association—In Advance—Nostrums.

